# Pregnancy Complications Can Foreshadow Future Disease—Long-Term Outcomes of a Complicated Pregnancy

**DOI:** 10.3390/medicina57121320

**Published:** 2021-12-01

**Authors:** Anca Maria Panaitescu, Mihaela Roxana Popescu, Anca Marina Ciobanu, Nicolae Gica, Brindusa Ana Cimpoca-Raptis

**Affiliations:** 1 Carol Davila Department of Obstetrics and Gynecology, University of Medicine and Pharmacy, 020021 Bucharest, Romania; anca.panaitescu@umfcd.ro (A.M.P.); anca.ciobanu@umfcd.ro (A.M.C.); gica.nicolae@umfcd.ro (N.G.); brindusa.cimpoca@yahoo.com (B.A.C.-R.); 2 Filantropia Clinical Hospital, 011171 Bucharest, Romania; 3 Cardiology Department, Elias University Hospital, 011461 Bucharest, Romania

**Keywords:** preeclampsia, cardiovascular risk, gestational diabetes, endothelial cells, heart failure with preserved ejection fraction, hypertensive disease of pregnancy

## Abstract

During gestation, the maternal body should increase its activity to fulfil the demands of the developing fetus as pregnancy progresses. Each maternal organ adapts in a unique manner and at a different time during pregnancy. In an organ or system that was already vulnerable before pregnancy, the burden of pregnancy can trigger overt clinical manifestations. After delivery, symptoms usually reside; however, in time, because of the age-related metabolic and pro-atherogenic changes, they reappear. Therefore, it is believed that pregnancy acts as a medical stress test for mothers. Pregnancy complications such as gestational hypertension, preeclampsia and gestational diabetes mellitus foreshadow cardiovascular disease and/or diabetes later in life. Affected women are encouraged to modify their lifestyle after birth by adjusting their diet and exercise habits. Blood pressure and plasmatic glucose level checking are recommended so that early therapeutic intervention can reduce long-term morbidity. Currently, the knowledge of the long-term consequences in women who have had pregnancy-related syndromes is still incomplete. A past obstetric history may, however, be useful in determining the risk of diseases later in life and allow timely intervention.

## 1. Introduction

**Figure 1:**
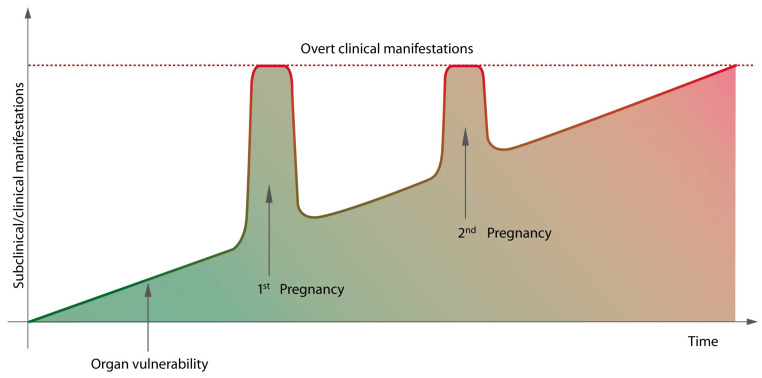
Organ vulnerability leading to transitory clinical manifestations during pregnancy, before overt, permanent clinical manifestation.

During gestation, the maternal body should increase its activity in order to fulfil the demands of the developing fetus as pregnancy progresses. Each maternal organ adapts in a unique manner and at a different time during pregnancy [1]. Physiological changes that healthy pregnant women face include a pro-atherogenic metabolic state [2,3], increased cardiac output [4], hypercoagulability [5] and high inflammatory response [6]. After mid pregnancy, insulin resistance [4,7] and hyperlipidemia [8] reach their peaks. After 24 weeks, pregnancy-related complications are more prevalent. These become apparent when the maternal organs are unable to compensate the demands that increase with an increase in gestational age. In an organ or system that was already vulnerable before pregnancy, the burden of pregnancy can trigger overt clinical manifestations. Maternal body activity decreases after delivery, and placental removal and symptoms may disappear. Some clinical features may reappear later in the woman’s life when her body reserves decrease. Age-related changes trigger a metabolic syndrome and pro-atherogenic state, leading to cardiovascular disease and diabetes mellitus (Figure 1). Therefore, it is believed that pregnancy acts as a medical stress test for mothers [9].

The aim of this paper is to review the current data on long-term outcomes after pregnancy complications based on the most recent meta-analysis and systematic reviews performed on this subject. We discuss possible interventions to decrease long-term morbidity and future perspectives. We also explore the possibility that screening models used in pregnancy not only identify pregnant women at risk of developing pregnancy-related syndromes but also a population that has a high risk of developing unfavorable outcomes later in life.

## 2. Preeclampsia and Future Cardiovascular Risks for Mothers

Preeclampsia (PE) is a pregnancy-related complication affecting multiple maternal organs characterized by new-onset hypertension after 20 weeks. In previously healthy pregnancies that are complicated by PE, pre-existing, subclinical cardiovascular risk factors are usually identified: maternal obesity [10], smoking, elevated lipid levels [11], hypertension [12], insulin resistance [13] and thrombophilia [14]. In patients who develop PE before 32 weeks, trophoblastic invasion is suboptimal, suggesting decreased uteroplacental blood flow [15]. High resistance in the uteroplacental flow is evident long before the clinical onset of high blood pressure. The poorly perfused placenta appears to be the source of anti-angiogenic factors that affect maternal vascular endothelium and fenestration of glomerular capillaries, causing proteinuria [16]. Women with high blood pressures in early pregnancy appear to be more sensitive to the circulating anti-angiogenic factor soluble fms-like tyrosine kinase-1 (sFlt-1) and, therefore, are at higher risk of developing PE [17]. First trimester screening for PE with accurate prophylactic measurements [18] and efficient treatment when hypertensive disorders are detected [19] can improve pregnancy outcomes and reduce the risk of long-term cardiovascular consequences. A vulnerability to future cardiovascular disease after PE has been described for many years [20]. Systematic reviews have put together numerous studies on the long-term consequences of PE and quantified the risk of future cardiovascular disease (CVD) after pregnancy with and without PE [21]. After one pregnancy is affected by PE, there is a 6 times increased chance of having a recurrent ischemic attack within a year after developing acute coronary syndrome [21]. Melchiorre et al. describe a so-called “dose-dependent” effect of hypertensive disorders of pregnancy (HDPs) and the long-term risk of developing chronic hypertension depending on the severity of PE, the onset of complications during gestation, the necessity of iatrogenic preterm birth, the association with intrauterine fetal growth restriction (FGR) and the number of gestations affected by HDPs [22]. After at least two pregnancies with PE, there is a 3 times increased risk of developing hypertension [23] as well as a shorter lifespan [24]; an increased chance of stroke (hazard ratio 5.10) [25]; an increased chance of ischemic heart disease (hazard ratio 3.3) [25]; and increased rates of heart failure, cerebrovascular accident and hospitalization because of cardiovascular disease [26].

**Figure 2:**
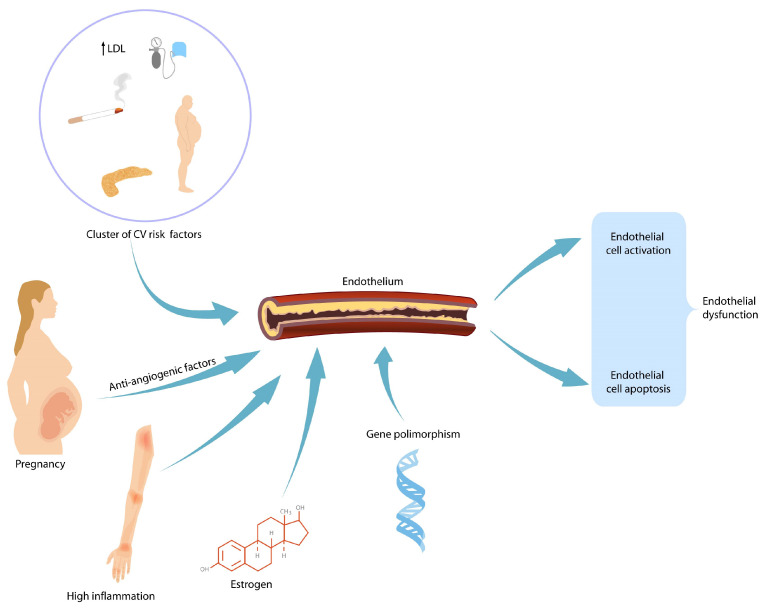
Endothelial dysfunction augmented by female-specific and non-specific risk factors. CV—cardiovascular; LDL—low-density lipoprotein.

In patients with HDPs, heart failure is more likely to develop within 5 years postpartum [27]. The associated risk of later CVD is lower for pregnancy-related hypertension than for PE but is still elevated (RR 1.9–2.5) [28]. Thus, HDPs and PE in particular may be regarded as novel risk factors for women’s future cardiovascular health, with a constant need for long-term follow-up. There are data linking PE and diastolic heart failure, but whether the latter is a consequence of the former, or whether there is a common pathogenic background, is still under debate [29,30]. Research into early changes in diastolic function and follow-up on markers common to both pathologies (N-terminal pro-B natriuretic peptide—NT pro-BNP, high-density lipoprotein cholesterol [30]) might be a worthwhile undertaking. The pathophysiological mechanism behind the increased subsequent risk of CVD is linked to endothelial dysfunction and structural abnormalities, including increased carotid intima–media thickness and accelerated coronary calcification and plaque deposition (Figure 2) [31]. This endothelial damage persists beyond the acute phase. However, whether this damage persists in the long term and is responsible for the increased number of cardiovascular events seen in this group is still up for debate. Preeclamptic pregnancies are characterized by increased CD 34+ cells as a reaction to endothelial dysfunction [32]. Whether the number of endothelial progenitor cells (EPC) can be linked to the severity of preeclampsia remains to be demonstrated.

**Figure 3:**
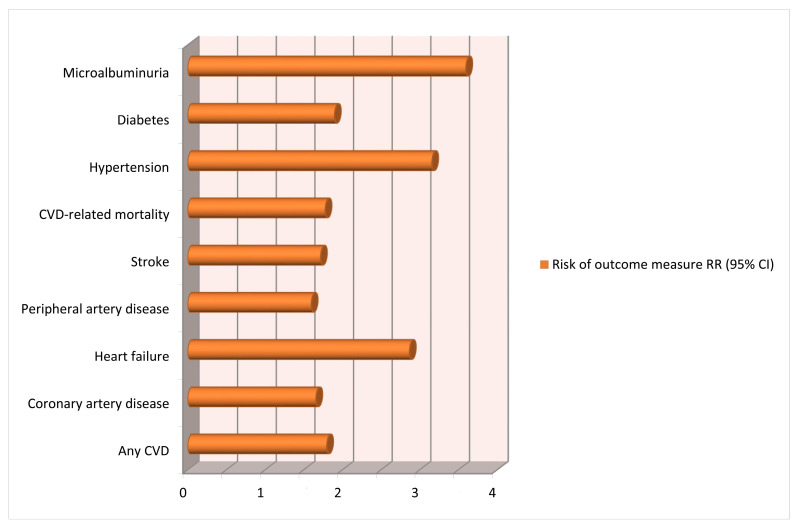
Risk of long-term cardiovascular consequences after a pregnancy complicated by hypertensive disorders in preeclampsia [19,20,33].

The latest meta-analysis included 73 studies (including cohort and case–control studies), analyzed over 13 million pregnancies and showed an important association between HDPs and long-term consequences on maternal cardiovascular system [33]. Important heterogeneity was caused by all or part of the variables, including type of exposure, follow-up time, geographic region and sample source. This is most probably caused by the results from the various reviews performed over the years. We acknowledge the limitations of the meta-analysis in terms of heterogeneity of the included studies and lack of long-term follow-up (Figure 3).

## 3. Preeclampsia and Future Renal Disease in Mothers

Preeclampsia is an endothelial disease characterized by high blood pressure with commonly coinciding proteinuria. Endothelial changes in renal glomeruli observed in pregnancies complicated by PE are reversible when glomerular scarring is not attained [34]. The reversibility of endotheliosis depends on cessation of the cause of endothelial injury. In the case of pregnancies with PE, fibrinous and granular deposits in the glomeruli persist for months after delivery and removal of the placenta [35]. During this time, microalbuminuria can still be detected at any glomerular filtration rate and acts as a cardiovascular disease marker. Preeclampsia alters renal function during gestation and also increases the risk of future chronic hypertension, chronic kidney disease (CKD) and cardiovascular disease [36]. Preexisting kidney vulnerability prior to pregnancy can explain both the appearance of PE and the long-term organ disease.

After one pregnancy complicated with PE, the mother has a fourfold increased risk of developing end-stage renal disease within 10 years after delivery [37]. After more than one preeclamptic pregnancy, a low-birthweight baby or a preterm birth increases the mother’s risk even further [37].

Accurate screening for PE, prevention or delay in the development of kidney disease in preeclamptic pregnancies will improve maternal long-term consequences. Albuminuria should be checked after birth to identify those mothers that need further monitoring of kidney function.

## 4. Gestational Hypertension and Future Cardiovascular Risks for Mothers

All HDPs are associated with increased risk of arterial hypertension even in the absence of pregnancy risk factors, such as obesity and smoking [38]. When compared to mothers who have late-onset or term preeclampsia, mothers who had gestational hypertension (GH), without proteinuria, have a similar risk of developing CVD and chronic hypertension [39]. Gestational hypertension is associated with a higher risk of kidney disease or diabetes mellitus [38].

In 2020, Garovic et al. showed that the total HDP burden expressed as incidence per-woman is considerably higher than that expressed per-pregnancy. By only looking at hypertension per-pregnancy, there is an underestimation of women who are affected by this condition and may be at risk of future heart or renal disease. Examining the per-woman rate allows a better assessment of women with more than one pregnancy, who may have had hypertension during one of the pregnancies but not the other [40].

## 5. Gestational Diabetes Mellitus

Usually, gestational diabetes mellitus (GDM) becomes apparent in the third trimester of pregnancy in patients who have pre-existing insulin resistance or have a reduced capacity to secrete insulin in response to gestational insulin resistance [41]. Universal GDM screening should be considered [42], as women who had GDM in a previous pregnancy are at increased risk of developing type 2 diabetes mellitus (DM) later in life. A meta-analysis of about 675,000 women that had follow-up for 28 years revealed that mothers with GDM have a seven-fold increased risk of developing type 2 diabetes [43]. It is therefore recommended that all mothers who have had GDM have glucose testing 6-12 weeks after delivery and every year thereafter [44]. Lifestyle advice, such as weight control, diet and exercise, should be included in the postnatal follow-up, as obesity in women with GDM triggers a greater risk of developing type 2 DM [45]. Further follow-up with regular assessment of glucose levels, blood pressure and timely intervention with metformin is beneficial in limiting the emergence of morbidity associated with type 2 DM [46].

Carr et al. first demonstrated in 2006 the link between GDM and future maternal cardiovascular disease risk [47]. Years later, studies quantified the risk, and the last meta-analysis in 2019 included almost 5,400,000 women and concluded that pregnant women with GDM have a twofold increased risk of cardiovascular events [48]. The American Heart Association (AHA) includes a prior history of GDM in the classification of cardiovascular risk factors in women [49].

In 2010, the Kidney Early Evaluation Program (KEEP) showed evidence that mothers with GDM have a greater prevalence of proteinuria when compared to women without DM [50]. In 2018, a Danish group conducted a 16-year follow-up for women with GDM and concluded that mothers with GDM were more likely to show a high estimated glomerular filtration rate (eGFR) 9–16 years after birth, which could suggest early stages of glomerular hyperfiltration and renal damage [51].

## 6. Thrombosis during Pregnancy

Physiological pregnancy is characterized by low-grade intravascular coagulation, and during pregnancy and puerperium, the risk of deep venous thrombosis (DVT) is six-times higher [52]. There is limited current knowledge of the long-term risks of venous thrombosis during pregnancy. Cohort studies researching long-term complications have not been conducted in this population, and the present evidence is based on data from a few observational studies [53]. Long-term generic quality of life and subjective well-being 3–16 years after a pregnancy complicated with thrombosis were not different from the general population. Mothers with post-thrombotic syndromes such as chronic venous congestion seemed to have poorer quality of life and an impaired general health [53].

Thrombophilia can be associated with a higher risk of PE [54]. Follow-up of women who have had PE showed that their risk of future thrombosis is twofold higher when compared to mothers who were normotensive during pregnancy [54].

## 7. Thyroid Disease in Pregnancy

Thyroid disease is the second most common endocrine disorder affecting women of reproductive age. Hyperthyroidism is present in 1 in 200 pregnancies. Symptomatic hypothyroidism is present in up to 1 in 100 pregnancies, and the incidence of subclinical hypothyroidism is much higher [55]. Approximately one in ten young women have anti-thyroid peroxidase (TPO) antibodies, and they are present in one in four euthyroid women who have a member of the family with thyroid disease [56]. Almost half of women with TPO autoantibodies develop postpartum thyroid dysfunction, which may present as hyper- or hypo-thyroidism. Almost half of all women who develop postpartum thyroiditis and who have TPO antibodies will remain hypothyroid for their entire life [57]. After a pregnancy with postpartum thyroiditis, there is a 70% risk of recurrence in a subsequent gestation [58]. Due to the strong association between TPO antibodies and future thyroid disease, clinical screening and checking thyroid function at least once during gestation, during puerperium or one year postpartum are recommended [59].

## 8. Liver Disease

Physiological pregnancy triggers cholestasis, and as pregnancy progresses, the maternal liver metabolizes high titers of steroid hormones and secretes them into bile [60]. Women with previously subclinical cholestatic disorders, including specific bile acid transporter defects, cholelithiasis, hepatitis C or cholangitis, are at increased risk of developing intrahepatic cholestasis of pregnancy (ICP) [61,62]. The increased risk of recurrence of ICP in subsequent pregnancies (up to 90%) suggests an underlying maternal cholestatic disorder that is overwhelmed toward term [63].

If hepatic function does not normalize postpartum, mothers with ICP should be assessed for subclinical hepatobiliary disease. The long-term complication for mothers with a history of ICP will depend on the underlying cholestatic defect. The most common conditions associated with ICP are hepatitis C and cholelithiasis [63], and they should have a proper screening. The acute fatty liver of pregnant women or those with HELLP syndrome (preeclampsia/hemolysis, elevated liver enzymes and low platelets) can cause notable liver disease [64]. Long-term survival of women with a history of hepatic failure due to these conditions can be achieved if the recovery from the multisystem complications of acute liver dysfunction is sufficient [64]. In 2019, Hämäläinen et al. conducted a study after 44-year follow-up of 571 mothers with ICP and concluded that survival is not changed due to ICP, but the risk of cholelithiasis, cholecystitis, pancreatic diseases and hypothyroidism is increased; therefore, long-term assessment is recommended for these women [65].

## 9. Possible Interventions to Decrease Long-Term Morbidity in Mothers with Pregnancy Complications

### 9.1. Preeclampsia

First-trimester screening for PE with the model of The Fetal Medicine Foundation offers the best predictive performance by combining maternal factors, mean arterial pressure, the uterine artery pulsatility index (UtAPI) and the serum placental growth factor (PLGF). It offers a detection rate of 90% for PE before 32 weeks, 75% for PE before 37 weeks and 41% for term PE at a 10% false positive rate [66]. This algorithm has undergone successful internal validation and calibration [67]. Offering aspirin 150 mg/day before 16 weeks of gestation successfully reduces the chances of developing PE [18]. Moreover, appropriate antenatal screening reduces the risk of severe adverse perinatal outcome associated with PE, as seen in a recent study [68]. We appreciate that the first-trimester screening actually identifies mothers that are at high risk of future cardiovascular disease later in life. If prophylactic intervention is successful and the pregnancy is not complicated by a hypertensive disorder, the mother’s risk of long-term CVD may be reduced, but it is not known whether it reaches that of the low-risk pregnancy population. In 2011. the American Heart Association added preeclampsia and delivery of a growth-restricted infant as pregnancy-related risk factors for cardiovascular disease [49], but what if we also consider first trimester screen-positive mothers as belonging to the same group?

Women with a complicated pregnancy could benefit from earlier cardiovascular disease prevention, referral to specialist care earlier in life and interventions such as lifestyle changes and exercise habit. It is recommended that both women and their healthcare provider are aware of the cardiovascular risk that follows after HDPs, and some suggest the monitoring of BP, renal functions and lipid profile yearly for the first five years after HDPs. In addition, lifestyle changes and control of CVD risk factors are beneficial [69]. The European Society of Cardiology guidelines recommend employing the N-terminal pro-B natriuretic peptide (NT-proBNP) as an investigative and monitoring tool for patients with hypertensive emergencies, including PE. NT-proBNP has been shown to be strongly related to cardiovascular events. Measurement of such biomarkers in women with prior HDPs needs further evaluation as a potential predictor or tool to risk stratify for future cardiovascular events [69]. Natriuretic peptide levels are associated with the occurrence of cardiac events, so an NT-pro BNP > 128 pg/mL in the 20th week of pregnancy is a predictor of subsequent events in pregnancy [70,71].

### 9.2. Future Perspectives in Understanding PE and the Long-Term Risks

The answers for the most important question of how to protect the endothelium lie in unraveling the mechanisms leading to this dysfunction. A recent study demonstrates increased levels of angiotensin II type 1 receptor auto-antibodies (AT1R-Ab) in women with PE both during pregnancy and as long as 4 years postpartum when compared to women with normal blood pressure during pregnancy [72]. These antibodies are a response to maternal inflammation and cause endothelial dysfunction both during pregnancy and postpartum and are associated with later development of hypertension, heart failure and diastolic dysfunction in late pregnancy. Hence, modulation of the angiotensin receptor might be a path to endothelial protection, at least postpartum. Another study connects low levels of soluble human leukocyte antigen G (sHLA-G) to modulation of the immune response toward the placental trophoblasts in preeclampsia. Surprisingly, in this study, sHLA-G levels were high for 3 years postpartum only for early-onset preeclampsia compared to normotensive pregnancies [73]. A recent review of modeling heart disease both in vivo and in vitro suggests that personalized models based on the patient’s own cells would prove to be the best strategy to better understanding the underlying pathological processes and testing new therapies [74].

Pravastatin has been used in pregnancy for the prevention of preterm and term PE in high-risk women [75,76]. Animal studies demonstrate that pravastatin treatment improved cardiac remodeling and output postpartum. Preeclampsia induces irreversible structural changes such as cardiac hypertrophy and fibrosis, which can be modulated by statins [77]. These structural changes can be linked to heart failure with preserved ejection fraction, and women represent the majority of this population [78].

The risk of developing PE in pregnancy can now be calculated for every pregnant woman, and prophylactic measures, such as aspirin or early interventions, can reduce the incidence of the clinical syndrome. We raise the question of whether the risk of long-term CVD is true also for patients that were found at high risk of developing PE when screening during pregnancy but, due to the prophylactic interventions, did not develop hypertensive disorders in pregnancy. Bearing this in mind, it could mean that screening algorithms employed during pregnancy not only identify women at high risk of developing PE but also CVD later in life. We acknowledge that future research is needed to determine whether the mothers that are screen positive for PE in the first trimester may benefit from statin medication after pregnancy to reduce the risk of CVD. Moreover, the inclusion of maternal endothelial dysfunction markers in the risk scores assessing the probability of PE may be a welcome addition to the current scores [31].

### 9.3. Gestational Diabetes

Preventive methods for the development of type 2 diabetes after gestational diabetes have been studied, and metformin is effective in preventing the occurrence of a metabolic syndrome and may have benefits in early treatment before weight gain or insulin resistance development. The American Diabetes Association recommends metformin use for women with prior gestational diabetes [79,80].

The postpartum follow-up, 6 weeks after delivery visit, gives the medical team, namely, the general practitioner, obstetrician and maternal–fetal medicine specialist, an excellent opportunity to raise awareness and provide advice on the long-term consequences of pregnancy complications as well as planning future follow-up (Table 1).

## 10. Conclusions

Pregnancy promotes a transient metabolic syndrome, similar to that which predisposes to atherosclerosis. Pregnancy complications such as gestational hypertension, preeclampsia and gestational diabetes mellitus put mothers at an increased risk of developing cardiovascular disease and/or diabetes later in life. Women that had such a pregnancy-related syndrome are encouraged to modify their lifestyle after birth by adjusting their diet and exercise habits. Blood pressure and plasmatic glucose level checking are recommended so that early therapeutic intervention can reduce long-term morbidity.

Other transient gestational syndromes result from the increasing demands of pregnancy unmasking the limited reserves of a vulnerable maternal organ. Currently the knowledge of the long-term consequences in women who have had a pregnancy-related syndromes remains incomplete. A past obstetric history may, however, be useful in determining the origin of diseases later in life and allow timely intervention.

## Figures and Tables

**Table 1:** Syndromes occurring in pregnancy, future maternal health-risks and possible interventions. HDPs—hypertensive disorders of pregnancy; PE—preeclampsia; GH—gestational hypertension; GP—general practitioner, family physician; OGTT—oral glucose tolerance test; DVT/PE—deep vein thrombosis/pulmonary embolism.

Pregnancy-Related Syndrome	Long-Term Maternal Risk	Possible Interventions
HDPs/PE/GH	Cardiovascular morbidity Diabetes Metabolic disturbances	Maternal education and awareness Lifestyle changes Smoking cessation GP regular follow-up Regular BP monitoring Regular metabolic profiling Aspirin Statins
Gestational diabetes	Type 2 diabetes	Maternal education and awareness Regular OGTT Diet and lifestyle changes Metformin
Cholestasis	Liver disease Cholelithiasis Pancreatic disease	Regular GP monitoring
Thyroid disease	Postpartum thyroiditis Hypothyroidism	Maternal education and awareness Regular GP monitoring Endocrinologist consultation
Thrombosis	Risk of DVT/PE	Awareness and reporting GP follow-up Medication
